# Withaferin-A Can Be Used to Modulate the Keratin Network of Intermediate Filaments in Human Epidermal Keratinocytes

**DOI:** 10.3390/ijms21124450

**Published:** 2020-06-23

**Authors:** Michael C. Keeling, Núria Gavara

**Affiliations:** School of Engineering and Material Science, Queen Mary University of London, Mile End Road, London E1 4NS, UK; m.c.keeling@qmul.ac.uk

**Keywords:** keratin, Withaferin-A, cytoskeleton

## Abstract

The mechanical state of cells is a critical part of their healthy functioning and it is controlled primarily by cytoskeletal networks (actin, microtubules and intermediate filaments). Drug-based strategies targeting the assembly of a given cytoskeletal network are often used to pinpoint their role in cellular function. Unlike actin and microtubules, there has been limited interest in the role of intermediate filaments, and fewer drugs have thus been identified and characterised as modulators of its assembly. Here, we evaluate whether Withaferin-A (WFA), an established disruptor of vimentin filaments, can also be used to modulate keratin filament assembly. Our results show that in keratinocytes, which are keratin-rich but vimentin-absent, Withaferin-A disrupts keratin filaments. Importantly, the dosages required are similar to those previously reported to disrupt vimentin in other cell types. Furthermore, Withaferin-A-induced keratin disassembly is accompanied by changes in cell stiffness and migration. Therefore, we propose that WFA can be repurposed as a useful drug to disrupt the keratin cytoskeleton in epithelial cells.

## 1. Introduction

Drug-based disruption of cytoskeletal (CSK) networks offers a rapid, dose-responsive and often reversible technique for studying mechanotransduction and cell signalling. While actin, myosin and tubulin all have unique sets of specific and well-characterised drugs to disrupt their organisation, there has been little effort to similarly identify drugs for disrupting intermediate filaments (IFs). Recently, IFs have emerged as important players in cells’ mechanical properties: they impart protection on the nucleus during migration [1] and are capable of withstanding high strains [2]. They are also biomarkers in certain types of cancer [3], and when mutated or altered in terms of expression or assembly, can lead to a unique set of diseases with aberrant tissue mechanics [4,5]. Therefore, identifying drugs that can disrupt IFs with little side-effects is a timely task.

It is important to note that IFs are varied: they consist of 72 proteins grouped into five types. Generally, agents to disrupt IFs are considered specific to one of these types. Keratins are cytoskeletal proteins made up of Type I and Type II IFs. They are found throughout the body in various epithelial tissues where they not only provide mechanical stability to cells but also are involved in signalling pathways, immune function and protein synthesis [6]. In the past, a number of drugs have been used to modulate keratin structure, each with certain drawbacks: Okadaic acid can cause large granules of keratin in the cytoplasm [7], and Acrylamide can cause disruption and bundling in the actin and tubulin networks [8,9]. While IFs of each type are distinct, the rod domain is highly preserved between them [10]. Therefore, drugs that are known to act on the rod domain in one type of IF are likely to be effective in other IFs.

One such drug is Withaferin-A (WFA), which is used to disrupt vimentin. It was put forth by Bargagna-Mohan [11] and is derived from the ayurvedic medicine Ashwagandha. At the time, it was known as a tumour suppressor and an inhibitor of angiogenesis. They showed that it bound Vimentin in the rod domain, at the C-237 residue, causing perinuclear aggregation of the filaments. As the WFA- bound filaments cannot be incorporated into the existing network, this mainly affects regions of high IF turnover. Since the rod domain is preserved in Keratin, WFA suggests itself as a potential disruptor of keratin assembly in a similar fashion as it disrupts vimentin assembly. It has in fact been shown to disrupt keratin in MCF-7 cells [12], but at potentially damaging and cyto-toxic concentrations above those typically used to disrupt vimentin. It thus remains to be verified whether at lower non-cytotoxic dosages, WFA can indeed be used as a keratin modulating agent for basic cell biology research.

Here, we report that in Human Epidermal Keratinocytes (HEKs), WFA disrupts keratin assembly at similar concentrations as those used for Vimentin disruption in other cell types. We detail the effect of WFA in doses between 0.5 and 3 µM on keratin organisation and Keratinocyte biophysical behaviour. We show that WFA can be of similar use in keratin-rich cell types, as previously shown in vimentin-rich cell types, thus opening up its re-purposing for studying Keratin filaments and expanding the arsenal of cyto-modulatory drugs.

## 2. Results

### 2.1. Cell Gross Morphology Is Not Affected by Routine Dosages of Withaferin-A up to 1 µM

For all experiments except migration, a timescale of three hours was chosen for drug incubation to be in line with previous work [12]. To visualise the HEK cells and their cytoskeleton, cells were stained for Keratin 14 (K14) (part of the dominant K14/K5 pairing in HEKs), α-tubulin and actin simultaneously. In addition, DAPI was also used to stain the cell nucleus.

To verify that the concentrations used did not lead to major cell cyto-toxicity, we monitored changes in nuclear area, cell spread area and overall cell gross morphology. For cell spread area quantification, we took advantage of the fact that all our cells were stained and imaged for f-actin (Supplementary Figure S1). Of note, actin was localised as a clear ring along the periphery of the cells and this organisation was not affected by WFA. Accordingly, we used the fluorescence images obtained from actin staining, in combination with those of keratin and tubulin to find the outline of the cell and quantify changes in cell spread area due to WFA treatment. Similarly, the DAPI channel was directly used to measure changes in nuclear area. In particular, we found that the cell spread area was only affected at 3 µM WFA, showing a significant drop of 56% (Supplementary Figure S2).

Similarly, the shape factor of the cells became slightly less rounded at 1 µM WFA and decreased by 23% at 3 µM WFA. Finally, the nuclear area only showed a significant drop of 25% at 3 µM WFA concentrations (Supplementary Figure S2), a decrease that has been shown by others to reflect the onset of cellular apoptosis [13]. Given that cellular and nuclear morphology did not display major changes until 3 µM WFA in HEKs, for the remaining experiments, we assumed 1 µM WFA to be a high but safe non-cytotoxic concentration to be used over the timeframe of three hours in HEKs. Accordingly, this is the concentration we chose for the live cell experiments moving forward.

### 2.2. Withaferin-A Modulates the Assembly of Keratin Filaments in a Dose-Dependant Manner

When imaged with epifluorescent microscopy, untreated primary human keratinocytes displayed a typical distribution of keratin, with a cage around the nucleus and filaments extending in a dense network to the cell periphery. Tubulin formed a similar dispersed network throughout the cell body while actin mainly existed in a ring at the cell periphery, with no large stress fibres crossing through the cell body or near the cell centre (Supplementary Figure S1).

**Figure 1:**
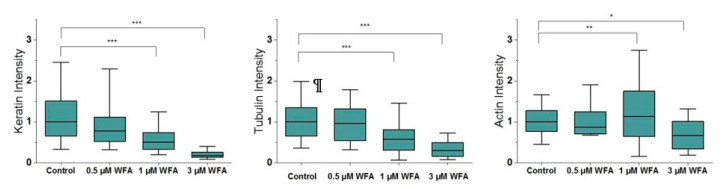
Normalised intensity of CSK filaments changes with increasing treatment of Withaferin-A (WFA) (*n* = 280). Box plots range from 25% to 75% with bars at 5% and 95%. Asterisks indicate a statistical difference (* *p* < 0.05, ** *p* < 0.01, *** *p* < 0.001, obtained using Dunnett’s test against control cells).

To quantify the effects of WFA on the cytoskeleton, we segmented filaments for each CSK network and quantified their intensity, density and angular variability per cell (Supplementary Figure S3). When we quantified keratin assembly, we saw a decrease in the fluorescence intensity of keratin filaments, starting at 0.5 µM WFA (Figure 1). This decrease was dose-dependent, with cells treated with 3 µM WFA reaching a 5-fold reduction in keratin assembled as compared to control cells. Importantly, this trend was preserved when we normalised our quantification of keratin assembly over cell spread area (Supplementary Figure S2j), indicating that our image quantification approach was robust and not impaired by associated changes in cell morphology and height. Furthermore, the density of the keratin network also decreased significantly starting at 1 µM WFA (Supplementary Figure S2d), suggesting that the WFA treatment did not lead to massive aggregation of keratin fragments in the cytoplasm, as observed for other drugs used to disrupt intermediate filaments.

As expected, given that a 3 h-long pre-treatment is likely to give rise to knock-on effects on other CSK network organisation [14], we also saw parallel dose-dependent changes in tubulin and actin assembly, even though they were smaller than those observed for keratin (Figure 1). Of note, while tubulin tended to disassemble following WFA treatment (by approximately 40% at 1 µM WFA), actin was observed to significantly increase at the highest non-toxic concentration for WFA treatment (by 13%), likely reflecting a finely-tuned interplay between the three cytoskeletal networks aimed at maintaining actin-based cell adhesion at the periphery of the cell.

Finally, the angular variability of cells is a measure of how aligned their filaments are, with a smaller variability indicating a more aligned network. We saw a consistent but small decrease in the angular variability of each CSK network at 3 µM WFA, indicating a decrease in the dispersion of fibres, potentially an effect of the decreasing shape factor of the cells (Supplementary Figure S2).

Together, these results show that WFA can be used to modulate primarily the amount and structure of filamentous keratin in human epidermal keratinocytes. Similar to what we previously found using other drugs to modulate filamentous cytoskeletal assembly [14], we observed some likely knock on and compensatory effects on the other cytoskeletal networks. It should be noted that cell sheets are much more resistant to WFA than single cells. In our hands, cells sheets could withstand high doses (3 µM) for over 24 h, as evident from the cell migration results (Supplementary Figure S4).

### 2.3. Withaferin-A Slows Keratin Filament Turnover

**Figure 2:**
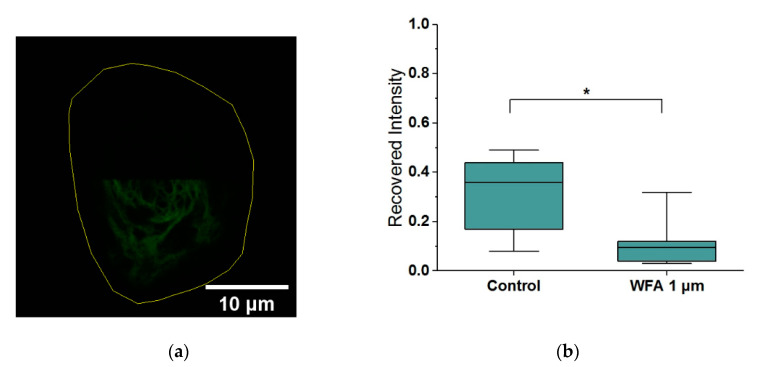
(**a**) Bleached cell and outline. (**b**) Withaferin-A limits FRAP recovery of GFP-Keratin in HaCaTs over 15 min (*n* = 12). Box plots range from 25% to 75% with bars at 5% and 95%. Asterisks indicate a statistical difference (* *p* < 0.05, obtained using Student’s *t*-test).

To assess whether changes in total assembly are linked to the changes in filament dynamics, as we have seen previously with vimentin [15], we carried out fluorescence recovery after photobleaching (FRAP) experiments using HaCaT cells expressing GFP-Keratin. In particular, we photobleached cells over half their area and then imaged them for 15 min (Figure 2). As expected, the recovery of control cells was not complete due to the high degree of bleaching and the relatively short time of imaging, reaching only 36% of their initial unbleached fluorescence. Cells which had been treated with 1 µM WFA displayed a significant 3-fold decrease in fluorescence recovery as compared to bleached control cells, thus indicating that WFA slows down the turnover of Keratin filaments.

### 2.4. Withaferin-A Increases Keratinocyte Stiffness

**Figure 3:**
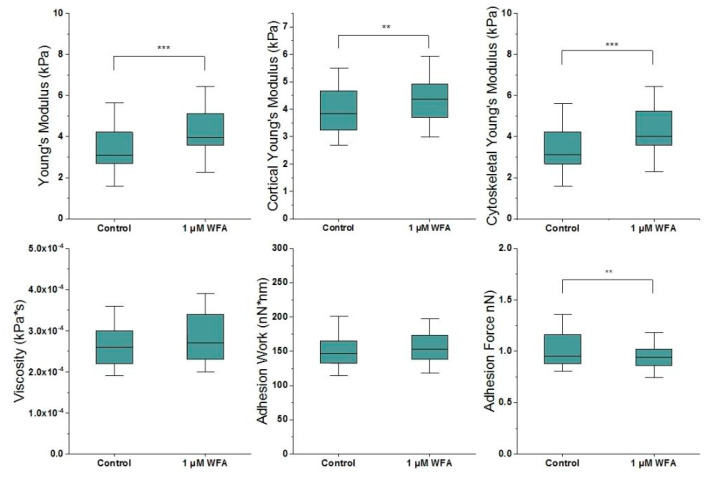
Withaferin-A stiffens HEK cells, specifically their cytoskeleton (*n* = 143). Box plots range from 25% to 75% with bars at 5% and 95%. Asterisks indicate a statistical difference (* *p* < 0.05, ** *p* < 0.01, *** *p* < 0.001, obtained using Student’s *t*-test).

Since keratin contributes to cells’ mechanical stability, we next assessed if the changes in filament organisation would lead to changes in cell mechanical properties. Sparsely plated HEKs were treated with 1 µM WFA, given we had identified that this causes disruption of the keratin network without causing widespread damage to the cell. The mechanical properties of treated and vehicle-treated cells were obtained using Atomic Force Microscopy (AFM)-based cell indentation. The measurements revealed that WFA increases cell stiffness by approximately 30% (Figure 3). It should be noted that the cell cortex primarily contains actin, while keratin is more dominant in the cytoplasm. Therefore, to further interrogate the role of keratin, we split the force curves into superficial (cortical) and deep (cytoskeletal) sections to better pinpoint the source of the stiffness change. On the one hand, there was a strong increase in cytoskeletal stiffness, suggesting that this is the main driver of the change in global stiffness identified above. On the other hand, we also found a smaller but significant increase in cortical stiffness, that parallels the increases in actin staining observed above (Figure 1). Of note, our two-dimensional (2D) image projections display actin as highly localised in the cell periphery, a tell-tale sign of a thin layer of cortical actin that is found throughout the cell’s apical surface [16]. Thus, our combined AFM an immunostaining results suggest that the cortical actin layer becomes thicker after WFA treatment, likely as a consequence of the interlinked nature of the CSK networks. Cellular viscosity, which intermediate filaments are thought to dominate, did not change with Withaferin-A treatment. We also measured non-specific adhesion work and force at the cell surface and saw a small but significant decrease in the adhesion force. Since this is at the cell cortex, it is likely effected by actin.

### 2.5. Withaferin-A Disrupts Keratinocyte Migration and Halts It at High Doses

Next, we sought to establish if the observed changes in keratin filaments and cell mechanical properties affected one of the key functions of epidermal cells: wound healing. Confluent cell sheets of GFP-keratin HaCaTs were wounded with a 10 µL sterile pipette tip and incubated with 0.5 to 3 µM WFA for 24 h. Given that complete wound healing was not achieved during the course of the 24 h, we chose to compute the changes in cell area coverage and normalised to the control cells. The results indicated a dose-dependent decrease in wound migration, with cells incubated with 3 µM not colonising any new area at all. These results are similar to those in Reference [11], which also show a dose-dependent reduction of migration in response to WFA in cells containing vimentin.

**Figure 4:**
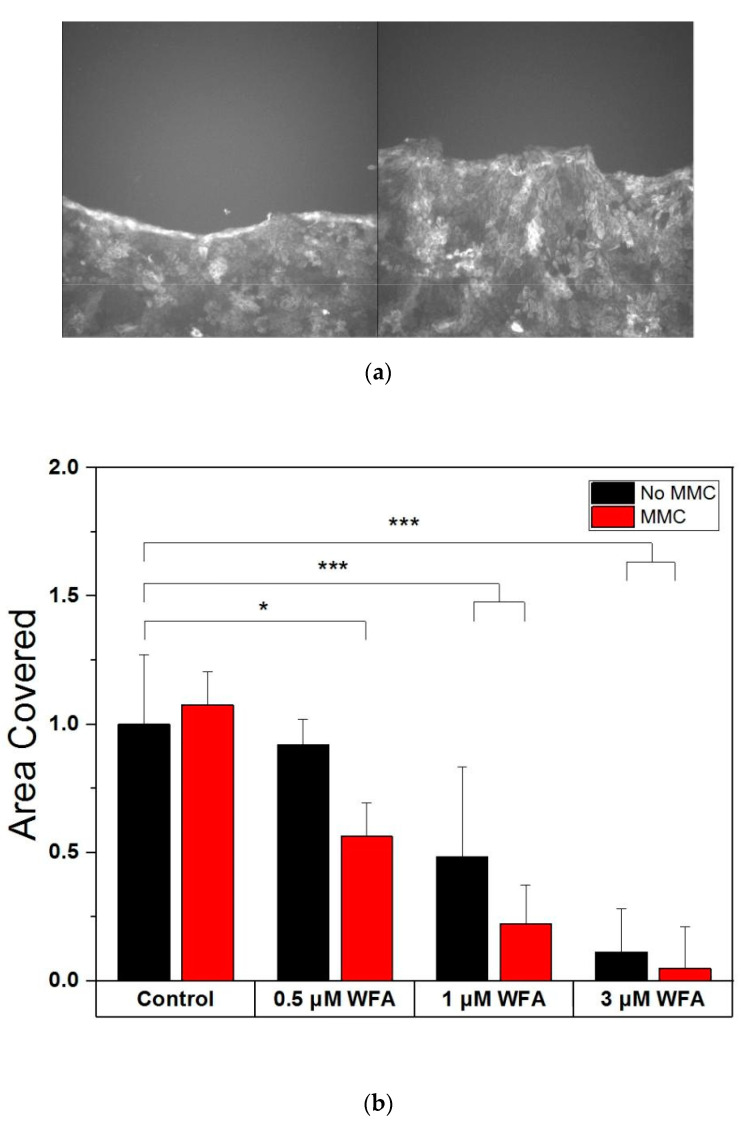
(**a**) GFP-keratin HaCaTs immediately after wounding and at t = 24 h (Control). (**b**) Withaferin A disrupts wound healing of GFP-Keratin HaCaTs in a dose-dependent manner (*n* = 61). Bar charts show mean and standard deviation. Asterisks indicate a statistical difference (* *p* < 0.05, ** *p* < 0.01, *** *p* < 0.001, obtained using Dunnett’s test against control cells not treated with MMC).

To verify that the decrease in sheet colonisation was a consequence primarily of WFA-impeded cell motility rather than reduced cell division, we carried out further experiments inhibiting cell division via incubation with 4 µg/mL Mitomycin-C (MMC) for 3 h before also being treated with WFA as above (Figure 4). Interestingly, there was no difference in the area reclaimed by cells with or without MMC treatment when no WFA was present, indicating that cell division was not a necessary driver of wound healing over 24 h. However, for all WFA dosages used, MMC-treated cell sheets showed reduced area recovery compared to their non MMC-treated counterparts (*p* = 0.0107). Cell sheets may compensate for the WFA caused loss of migration by relying on cell division for wound healing, which is why MMC treatment has an effect in this case. The lower migration of WFA-treated cells in the presence of MMC implies that WFA mainly targets migration, and not cell division, as if it had blocked division, the addition of MMC would have had no effect. Overall, the results show that WFA inhibits wound healing, specifically through targeting cell migration.

While epidermal keratinocytes normally do not express vimentin, wound healing is a special case where they temporarily can, and in the case of colony growth, it has been shown to be important for migration [17]. Since WFA preferentially disrupts vimentin in cells that contain both it and keratin [12], the possibility arises that the slowdown of cell-migration by WFA actually acts through vimentin disruption, not keratin. To check if that was the case, we stained wounded GFP-Keratin HaCaT cell sheets at 24 h of migration with vimentin antibodies and found no sign of vimentin filaments. Conversely, the leading edge of the migrating cells did appear to have altered keratin organisation when treated with WFA, and to a lesser extent, so did the main body of the cell sheet. This indicates that WFA’s action is likely only being applied to keratin in our experiments in HEKs cells.

## 3. Discussion

We showed here that in Human Epidermal Keratinocytes, keratin is disrupted by Withaferin-A. In particular, WFA’s effect on keratin slows the turnover of filaments and decreases the density of filaments in the cell. Cells become stiffer and their ability to heal wounds is slowed down or even completely stopped. No large non-filamentous aggregates are formed, meaning the filaments are still able to be quantified through microscopy. Importantly, the response is dose-specific, allowing for an adjustable response from minor disruptions of the network to a full-blown collapse.

It is worth stressing that the changes seen in tubulin and actin in response to WFA treatment are expected, based on our own previous work and the work of others. In detail, the CSK networks are intimately linked [18,19] and changes in tubulin or actin organisation have been seen before in WFA-mediated IF disruption [12]. Similarly, the interconnectedness of the cytoskeletal networks is also reflected on the mechanical role they play in cellular state. The increase in cell stiffness after WFA treatment is intriguing and only partially due to increases in cortical actin assembly. At the same time, it is reminiscent of the reinforcement in cell mechanics reported by others after treatment with drugs known to depolymerize the microtubules network [20,21,22]. While WFA has also been shown to covalently bond to cysteine residues on tubulin [23,24], its effects only occur at higher concentrations and incubation times compared to what we reported here. We thus conclude that the changes in tubulin we see are driven by the disruption of keratin. Nevertheless, future work could aim at assessing and comparing the different timeframes of filament disruption for keratin and tubulin due to WFA treatment. Further, while previous work has shown WFA’s ability to disrupt keratin, this has only been shown at very high concentrations, likely leading to cell damage [12]. Therefore, our work, which focuses on concentrations previously shown to be useful and non-cytotoxic in vimentin-rich cells [11], opens up the use of WFA as a keratin modulating agent in keratin-rich cells, such as HEK cells.

A mechanism of action between WFA and keratin has not been shown so far. In the particular case of vimentin, WFA is known to bind to the cysteine-327 residue, and when this residue is lacking, very high doses are needed to cause disruptions. The region of the cysteine-237 residue is the 2B region of the central rod domain, which is highly conserved in all intermediate filaments [10]. In keratin, the cysteine residue C-367 is known to be important for the organisation and assembly of the network [25], and it also sits in the 2B region. While the exact mechanism of action between WFA and keratin is outside of the scope of this paper, the similarity in dose and observed effects between vimentin and keratin suggest that the effect may be on this shared site.

The survival of cell sheets treated with high doses of WFA as compared to single cells shows that dosing of the drug needs to be considered in a broader context. In cell sheets, the keratin filaments near the cell boundary are anchored to cell–cell junctions and are more stable than in the edge of single cells, where they have a high turnover, as it is the nucleation point of their inward flux [26]. As turnover of keratin is lower in cell sheets, there are fewer opportunities for filament disruption, making the drug less effective. This is supported by our wound healing experiments, where the cells at the edges of cell sheets, who have fewer neighbours, appear to have more disrupted keratin. At the same time, other effects of WFA that are not intermediate filament-related may still occur at their normal rate, decreasing the suitability of WFA as a targeted IF disrupting drug at the tissue scale.

Drug-based and knockout-based approaches to removing CSK networks may not necessarily yield the same results. Cells from gene-knockout lines experience a complete loss of the target protein and may also engage compensatory mechanisms to adapt to the missing network. Drug-based disruptions are typically rapid, and their aim is not the removal of the building blocks of the network, but rather the inhibition of their assembly into larger polymeric structures. Multiple papers [5,27] show that Keratin knockout keratinocytes are indeed softer than their wild-type counterparts. Since our WFA-treated cells are stiffer, this may highlight the difference between drug-based and knockout-based approaches: in WFA-treated cells, keratin is not fully removed and may in fact be aggregating into stiffer structures over the period of treatment. Of note, we have also seen stiffening in vimentin-rich fibroblasts at high doses of WFA [15]. This difference however may only occur for certain aspects of cell mechanics: for cell migration, our drug-based modulation of keratin matches the behaviour of knockout cells. In mesenchymal cells, IF knockout slows cell migration, an effect that is thought to act through controlling cell–cell junctions and distributing forces in the leading edge [28]. Keratin’s association with desomosomal and hemidesmosomal junctions is one route of its control over cell migration. Desmosomes maintain tissue integrity and form at the front of cell sheets during migration. Keratin loss then causes unstable formation of desmosomes [29] which may lead to issues with migration. It is also important to note that the effect of high doses of WFA on migration begins upon drug addition, indicating a rapid mechanism of action.

The key role of keratin in processes such as wound healing or epidermal barrier function makes it an important target of study. Research on keratin will likely benefit from the use of WFA, since the ability to quickly modulate its organisation in a dose-dependent manner allows for a detailed interrogation of its properties and effects on cellular processes. WFA also suggests itself as a potential new drug against conditions which present excess of keratin assembly, such as hyperkeratosis.

## 4. Materials and Methods

### 4.1. Cell Culture

Primary human epidermal keratinocytes (HEKs) derived from human foreskin were a gift from John Connelly of the Blizzard institute. HEKs were co-cultured with J2 3T3 Swiss Albino murine fibroblasts as per Rheinwald and Green [30,31]. All HEKs used in this work were between passages two and six. Where live cell imaging of keratin was required, immortalised HaCaT cells expressing GFP-Keratin were used instead, also a gift from the Connelly group.

Culture media for fibroblasts when cultured alone was high-glucose DMEM with 10% Foetal Bovine Serum (FBS) and 1% Penicillin Streptomycin. For co-culture, this base medium was further supplemented with Cholera toxin (10^−10^ M), Epidermal Growth Factor (10 ng/mL), Hydrocortisone (0.5 µg/mL), Adenine (1.8 × 10^−4^ M) and Insulin (0.5 µg/mL). This supplemented media is referred to as FAD media.

For imaging experiments, HEKs were plated on dishes or glass coverslips coated in collagen (0.3 mg/mL) in FAD media for 24 h at a seeding density of 2500 cells/cm^2^. For experiments with live cells outside an incubator (AFM), cells were immersed in media with 25 mM HEPES buffer added. HaCaT cells were cultured and in the same media as J2 cells.

### 4.2. Drugs

Withaferin-A was sourced from Sigma/Merck as a powder. Stock solution was prepared in DMSO to 21.1 mM concentration and stored at −80 °C. Stock was aliquoted in 4.7 µL doses and stored at −20 °C in preparation for experiments. To standardise, for all experiments, working solution was created by diluting the 4.7 µL of 21.1 mM WFA in 1 mL of media to get 1 mL of 100 µM of working solution. The final concentration of DMSO in all experiments was always below 0.015%. Control cells were supplemented to match the highest DMSO concentration in the experiments.

Mytomycin C was sourced from Sigma/Merck, prepared to a stock solution of 0.4 mg/mL in PBS and stored at −20 °C. Treatments were performed for 3 h at 4 µg/mL.

### 4.3. Immunofluorescence Staining

For immunofluorescence staining, once cells reached 3 h of WFA incubation, they were washed in warm PBS, then fixed in 37 °C 4% PFA for 10 min and finally washed 3 times in PBS. Next, they were permeabilised with 0.25% Triton-x for 5 min and washed three times. Following this, they were blocked in 10% BSA for 1 h at room temperature, incubated with primary antibodies Keratin-14 (Santa Cruz, CA, USA, sc-58724), α -tubulin (, Cambridge, UK, ab4074), at 1:200, or Vimentin (Santa-Cruz, CA, USA, sc-32322) (1:50 to account for the increased number of cells in sheets) in 1% BSA overnight at 4 °C and washed 3 times in PBS (we have successfully used this vimentin antibody in other cell types to image filaments). Next, they were incubated with Alexa Fluor secondary antibodies (488/555), DAPI (Sigma) and phalloidin 633 (Santa Cruz, CA, USA) (all 1:1000) for 1 h at room temperature. After washing in PBS three times, slides were mounted using Prolong Glass. The mounting media was allowed to cure for at least 24 h before use.

### 4.4. Epiflourescent Imaging and Quantification

Cells were imaged with a Leica DMI8 epifluorescent microscope at 63X (Oil) magnification. Z-stacks with an interval of 0.2 µm were taken and reduced to a single image using an extended depth of field algorithm, allowing for fibres from the whole volume of the cell to be represented in one image. Cells’ area and shape factor (circularity) were calculated from the masked actin image. The equation for the shape factor is:

SF = 4*p*Area/Perimeter (1)

Images were processed through a filament analysis pipeline using code adapted from Gan et al. [32], which centres around the use of a steerable filter for finding and quantifying fibres in an image (Supplementary Figure S3). From the segmented fibre images, we can then extract parameters describing the keratin network: total intensity refers to the sum of intensity (background corrected) within the detected filaments. Density is a measure of proximity of filaments measured by blurring the filter mask using a disk-shaped filter which results in a self-proximity map of the network, then taking the mean of the map within the cell boundary. Results were pooled from two experiments.

### 4.5. FRAP

A pre-bleach image of the cell was taken in one plane. An area covering half the cell was bleached with 100% intensity for 5 cycles at a rate of 1 cycle per second. Following this, images were taken regularly over 15 min in the same plane of focus as the bleaching. Images were taken with the laser at 2% intensity, a pinhole of 1 AU and a pixel dwell time of 1.27 ms for 512 by 512 pixels. Images were processed in ImageJ manually. The change in the bleached section over fifteen minutes was divided by the difference in the bleached and unbleached section immediately after bleaching to measure the percentage of fluorescence recovered. Since the bleaching also reduced intensity in the unbleached portion of the cell, the percentage of fluorescence recovered in the bleached section was corrected for the recovery in the un-bleached section. Results were from a single experiment.

### 4.6. Atomic Force Microscopy

Cells were plated in collagen (0.3 mg/mL)-coated petri dishes for 24 h and placed in a petri dish heater on the AFM stage in 25 mM HEPES-containing media, for a maximum of one hour. Cells incubated with Withaferin-A were measured between 2.75 and 3.25 h of incubation with the drug. The AFM used was a JPK NanoWizard 4. Bruker MSNL-10 cantilevers with a nominal spring constant of 0.03 N/m were used with a setpoint of 3 nN. Measurements were taken in QI mode with a 300 µm/s cantilever speed over a grid of 32 by 32 pixels that covered the whole area of the cell. Data was processed using an in-house MATLAB pipeline which uses a contact point detection method optimised for adherent cells [33,34]. Since we found a large variability between cell mechanical properties between experiments on different days, results were from a single experiment designed to maximise cell numbers captured in one day.

### 4.7. Cell Migration

Wound healing experiments were performed with the Lumascope 720 microscope at 10× magnification. Densely seeded GFP-Keratin HaCaT cells were allowed to form a cell sheet. Next, they were wounded in the pattern of a cross using a 10 µL pipette. Cells debris were washed, and drugs were added at this point. Multiple fields of view were recorded for each wound. The first and last images were compared, and the area not covered by cells was manually marked on both of them. The difference in covered area between the two images gave the colonised area over 24 h. Results were pooled from two experiments.

## Supplementary Materials

Supplementary materials can be found at https://www.mdpi.com/1422-0067/21/12/4450/s1, Figure S1: Individual CSKs and merge for control and 0.5–3 µM WFA; Figure S2: a–c CSK Intensity Normalised by Cell Area changes with WFA, d–f, CSK Network Density changes with WFA, g–l, CSK angular variability changes with WFA, j, Cell Area, k, shape factor and l, Nuclear Area change with WFA; Figure S3: (**a**) Keratin Intensity image, (**b**) result of filament segmentation, (**c**) Self Proximity score using a disk-shaped filter. The mean of the score within the cell boundary gives the network density; Figure S4: HaCaT cells with GP-K14 (left, exposure time = 0.14 s) stained for vimentin (right, exposure time = 1.8 s. Magnification 20×.

## Abbreviations

WFA	Withaferin-A
HEK	Human Epidermal Keratinocyte
AFM	Atomic Force Microscopy
MMC	Mitomycin C
