# Supplementary material for: Withaferin-A Can Be Used to Modulate the Keratin Network of Intermediate Filaments in Human Epidermal Keratinocytes
Source: Int J Mol Sci. 2020 Jun 23;21(12):4450. doi: 10.3390/ijms21124450 (PMC7352337; doi:10.3390/ijms21124450)
Supplement: Supplementary file 1 [file ijms-21-04450-s001.pdf]

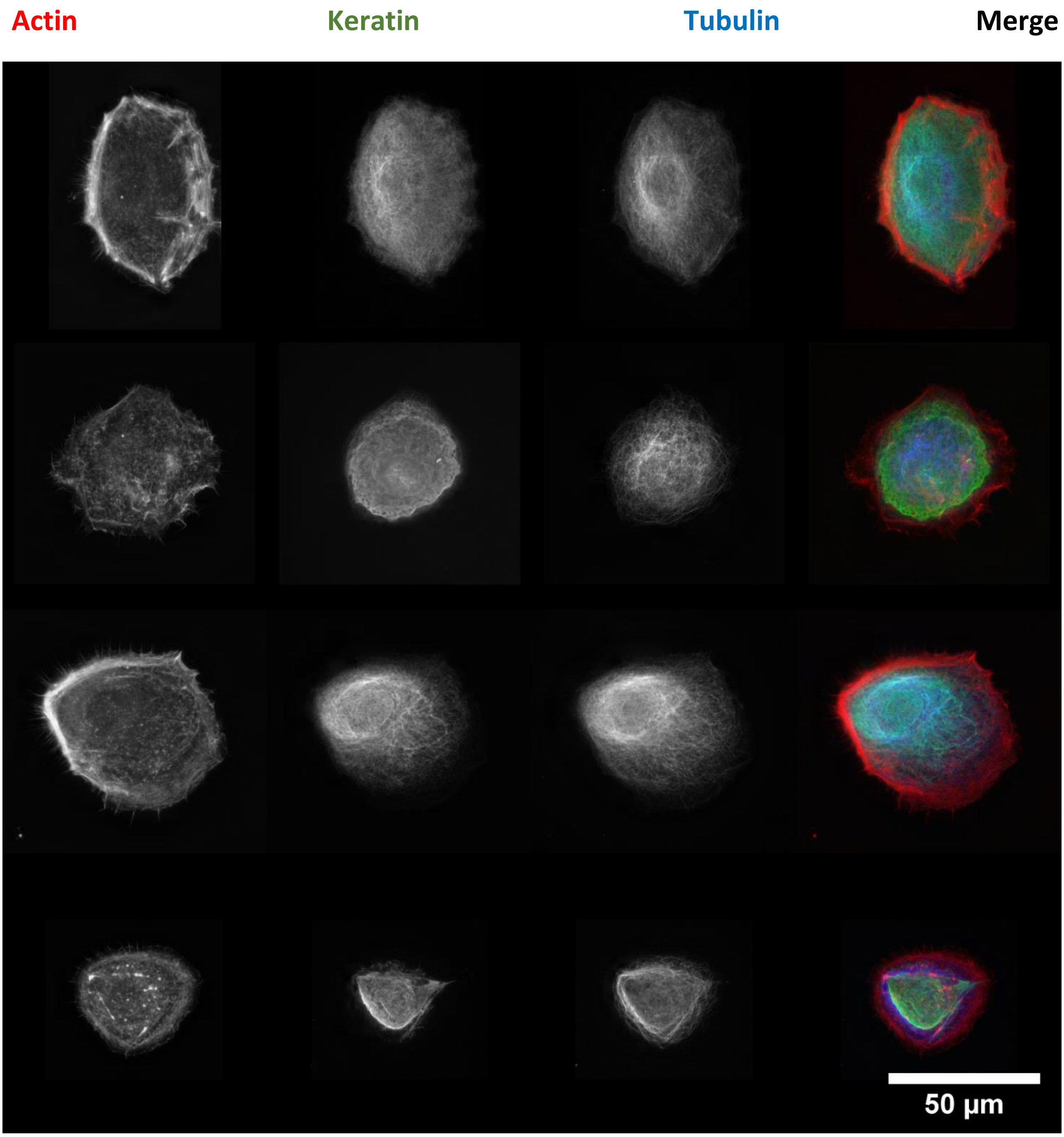

Figure 1. Individual CSKs and merge for control and 0.5-3 μM WFA.

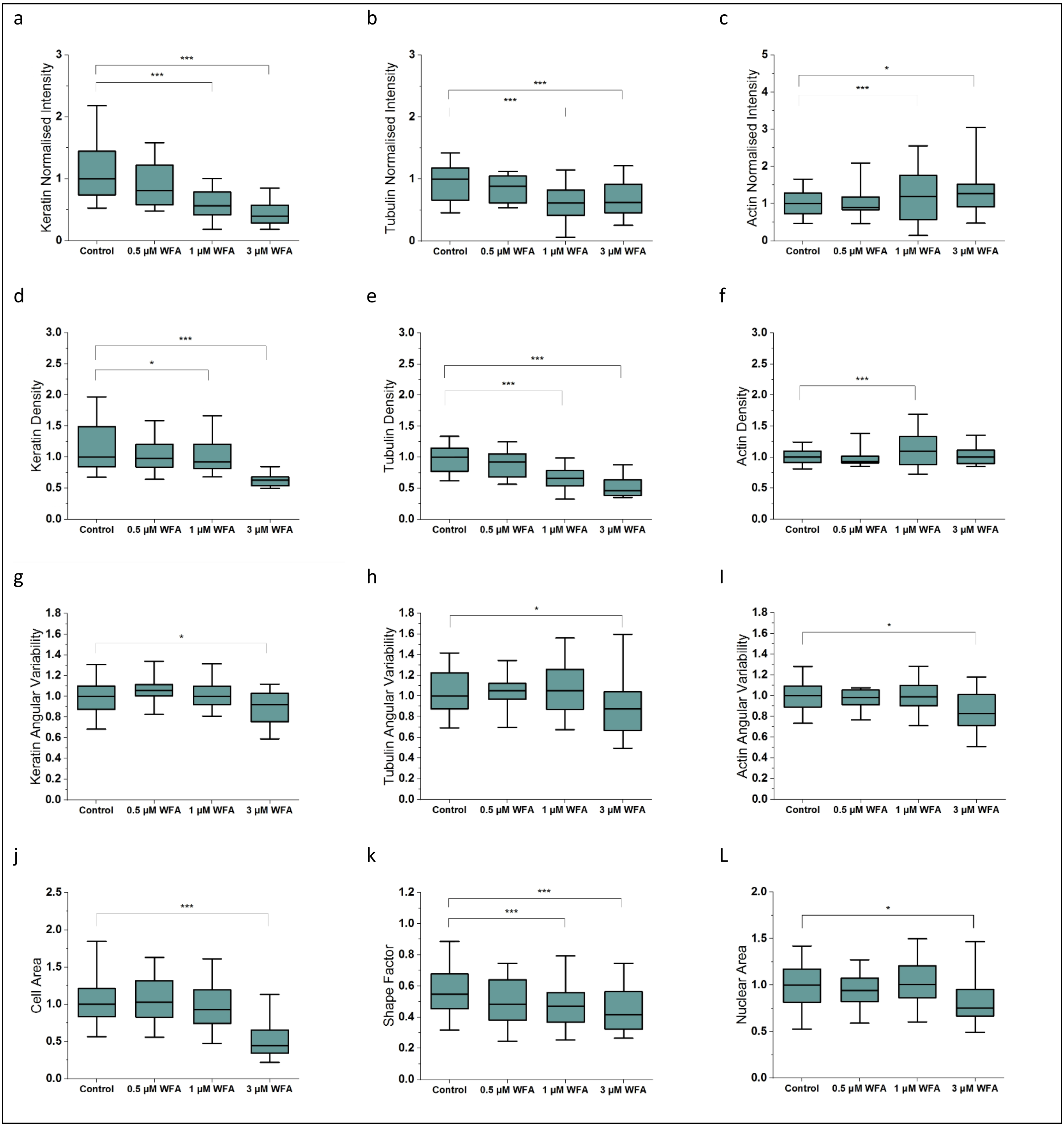

Figure 2. a-c CSK Intensity Normalised by Cell Area changes with WFA, d-f, CSK Network Density changes with WFA, g-l, CSK angular variability changes with WFA, j, Cell Area, k, shape factor and l, Nuclear Area change with WFA.

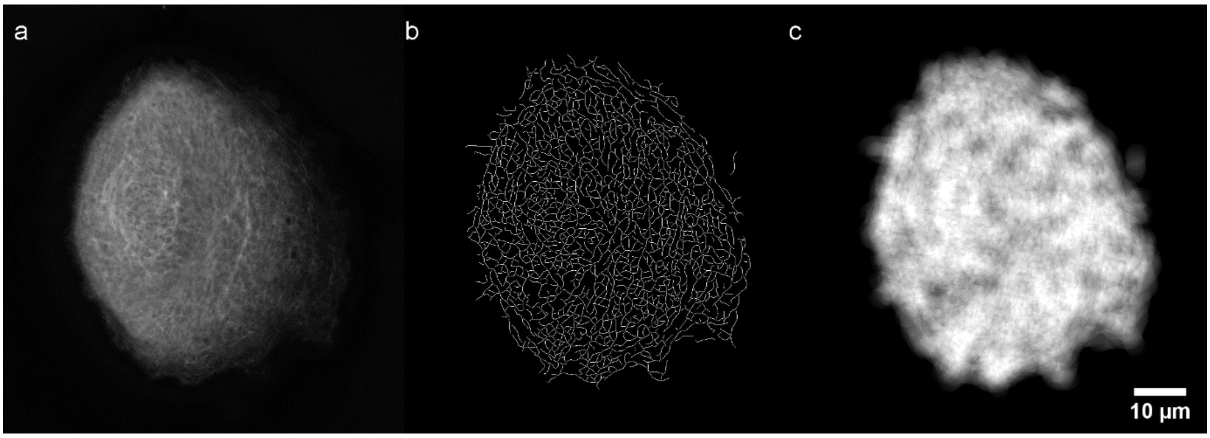

Figure 3. (**a**) Keratin Intensity image, (**b**) result of filament segmentation, (**c**) Self Proximity score using a disk-shaped filter. The mean of the score within the cell boundary gives the network density.

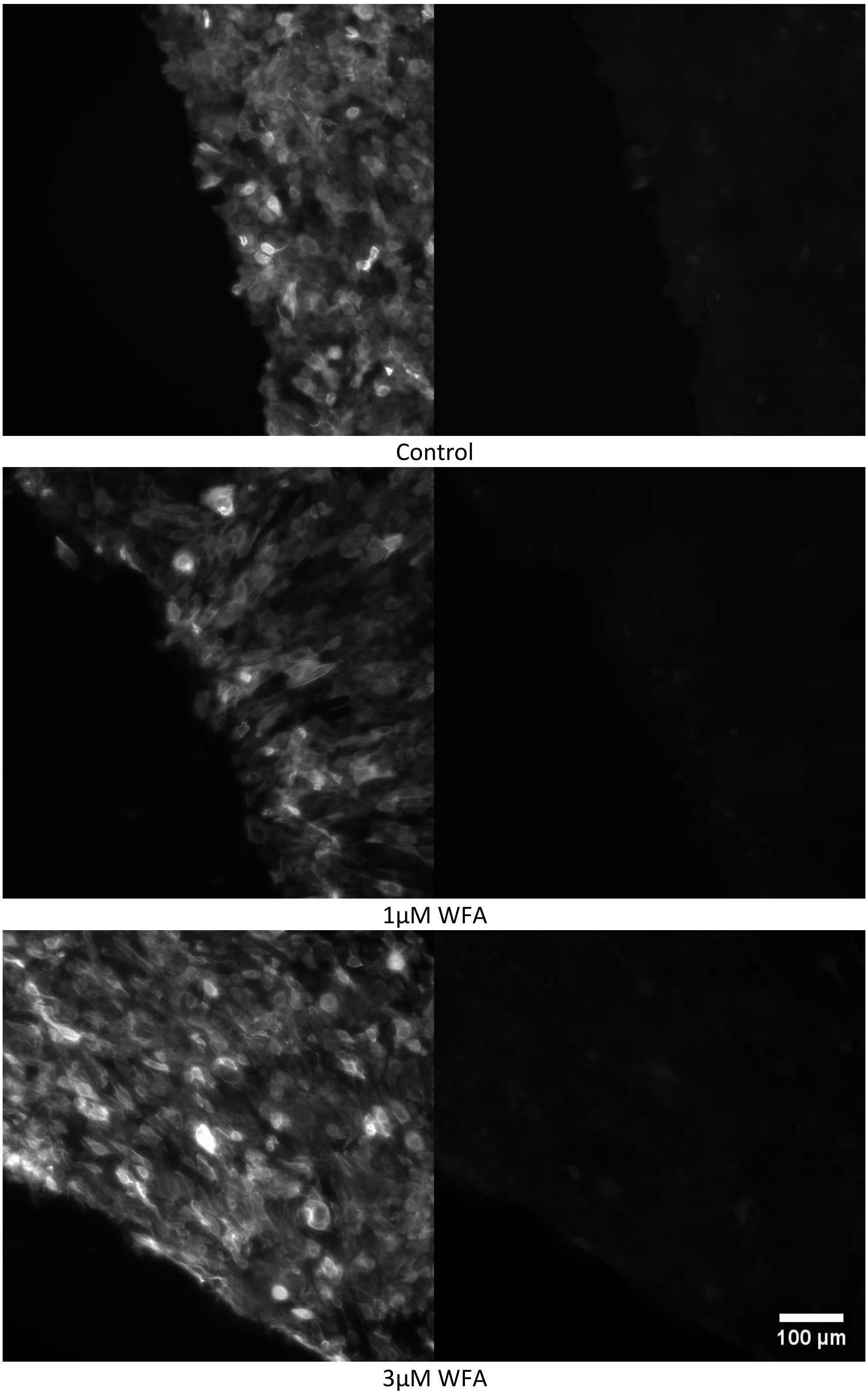

Figure 4. HaCaT cells with GP-K14 (left, exposure time=0.14 seconds) stained for vimentin (right, exposure time=1.8 seconds. Magnification 20x.
